# “It’s just in that sea of things that I never cared about”: perception of hepatitis B amongst university students in Aberdeen, North-East Scotland

**DOI:** 10.1186/s12889-019-6654-z

**Published:** 2019-03-21

**Authors:** Emma L. Davies, Shona Fielding, Gillian Noble, Emmanuel Okpo

**Affiliations:** 1 Public Health, NHS Grampian and University of Aberdeen, Summerfield House, 2 Eday Road, Aberdeen, AB15 6RE UK; 2 Institute of Applied Health Sciences, NHS Grampian and University of Aberdeen, Foresterhill, Aberdeen, AB25 2ZD UK; 3 Leith Mount Surgery, Edinburgh, EH6 4EG UK

**Keywords:** Hepatitis B, Blood borne virus, University students, Knowledge, Awareness, Perception, Risk, Education

## Abstract

**Background:**

A significant proportion of international students at UK universities are from regions with medium to high hepatitis B prevalence rates. Understanding the perception of students regarding hepatitis B infection is crucial for the development of appropriate information and services for this population group.

**Methods:**

Twenty semi-structured interviews were conducted with students from the University of Aberdeen. The following key areas were covered: knowledge, awareness, practices including testing, cultural and social aspects and general attitudes to health information and services. Interviews were transcribed verbatim and coded using a framework analysis approach.

**Results:**

The participants acknowledged hepatitis B to be a serious disease yet did not consider themselves to be at risk. They felt able to go to their General Practitioner if concerned about hepatitis B but emphasised that there was no indication that this was required. There was a general lack of knowledge about the disease including confusion over other types of hepatitis. This was linked to the perceived lack of attention given to hepatitis B in, for example, sexual health education and disease awareness raising campaigns. The participants expressed a desire for information on hepatitis B to be relevant to the student population, easy to understand, socially acceptable and easily accessible on student portals and social media platforms.

**Conclusions:**

Our study suggests that students in Aberdeen, North East Scotland lack knowledge and awareness of hepatitis B and do not perceive themselves as being at risk of hepatitis B infection. There is a need for more tailored hepatitis B messages to be incorporated into a range of contexts with clearer risk communication for the student population.

## Background

Hepatitis B virus (HBV) is a major health problem with an estimated 257 million people chronically infected globally [1]. The virus is transmitted through contact with infected blood or other bodily fluids such as saliva and semen. Prevalence rates vary considerably across different regions of the world; with prevalence rates of up to 6.2% reported in parts of South East Asia and Sub-Saharan Africa [1]. Scotland is classified as a low prevalence country with an estimated 0.1% of the population infected [2]. However, in certain sub-populations, such as the migrant community, the rates appear to be much higher. In Grampian, North East Scotland, around 50 cases of HBV are diagnosed every year; most of which are newly diagnosed chronic infections in migrants from high prevalence countries. Approximately 30% of University of Aberdeen students are international students and 20% are from high or intermediate HBV prevalent regions. Furthermore, most university students are young adults and are more likely to engage in behaviours and lifestyles that increase the risk of hepatitis B infection [3, 4].

Prompt diagnosis and appropriate management of cases of hepatitis B including effective contact tracing is necessary to reduce the risk of transmission of the infection. In a 2017 US study which included HBV testing, 74% of those with chronic HBV were previously unaware of their status and were not actively seeking to prevent spread to others [5]. Other population based surveys in low HBV prevalence countries such as the US and Netherlands have demonstrated consistently low levels of HBV knowledge amongst migrant communities [6–8] and lower levels of knowledge have been associated with lower testing rates [6, 7, 9].

The importance of acceptability, accessibility and perceived relevance on service utilisation has previously been highlighted [7, 9, 10]. Hence, understanding the knowledge, awareness and behaviours of specific groups, such as university students, are essential. By doing so, evidence-based targeted information and services can be developed and provided. Despite this, previous studies looking at the needs of students have relied heavily on quantitative methods alone and have tended to focus on students training to be healthcare professionals [11–14]. We have found no other study conducted in a UK context.

The aim of our study was to explore the HBV knowledge, awareness and associated behaviours of university students in Aberdeen, North East Scotland.

## Methods

### Design and setting

We report here the qualitative component of a larger mixed-methods study. The quantitative aspect was an online survey. Both undergraduate and postgraduate students were recruited from the University of Aberdeen, which has a total student population of around 14,000.

### Recruitment

All students registered at the University of Aberdeen in the 2015/16 academic year were emailed a link to the quantitative aspect of the study (the online survey). They were asked to provide contact details on completion of the survey if they wished to also take part in the interviews. Students were further made aware of the opportunity to be interviewed through posters on university notice boards and announcements in lectures. They were asked to contact the researchers through the study’s email address. Everyone who expressed a willingness to take part was followed up with an interview and there were no participants who dropped out at any point in the process.

### Interviews

Twenty interviews were conducted between 23 November 2015 and 22 February 2016 by one female researcher (EA; see *Acknowledgements*) who was employed by the University of Aberdeen to undertake this work. The researcher had experience in qualitative methods (interviews, focus groups, observing practice methods) but no personal or professional experience regarding HBV. The quantitative survey was conducted and completed prior to the interviews commencing. Participants were informed at the stage of recruitment that the interviewer would be female but did not know any further details about her. The first time they met was when the interviews were carried out. The interviews were conducted face to face, in a meeting room booked for the occasion on either of the two university campuses. Only the researcher and participant were present. An information sheet explaining the study was provided and written consent obtained from each participant before the interview began. It was explained that the purpose was to find out more around HBV knowledge levels in the student population.

The interviews were semi-structured; framed around the questions specified in the topic guide but with freedom to explore ancillary topics as the conversation progressed. The interviews generally lasted for twenty to thirty minutes. No participant was interviewed more than once. Perspectives were sought from men and women of a variety of nationalities, from undergraduate and postgraduate courses across all three University of Aberdeen colleges (Arts and Social Sciences, Life Sciences and Medicine, Physical Sciences.)

### Topic guide

The topic guide was directed by relevant previous literature [6, 9, 10] and expert opinion. It covered the following areas: knowledge, awareness, practices including testing, cultural and social aspects and general attitudes to health information and services. It was not piloted but the phrasing of some questions was modified as the interviews progressed and others tailored to the participant. This is in line with the iterative process of qualitative data collection and analysis [15]. Field notes were made after each interview and the resulting reflections used to alter the emphasis of further interviews in order to better explore emerging themes.

### Analysis

Interviews were audio-recorded, then transcribed verbatim either by the researcher (EA) or through the use of a confidential transcription service, NJC Secretarial. Transcripts were not able to be returned to participants for comment but the initial researcher (EA) and a further researcher (ED) independently checked them for accuracy against the audio recording.

Two researchers independently coded the data (EA and ED) through a framework analysis approach [16]. QSR International Nvivo and Microsoft Excel were used. Data immersion was achieved by reading through the transcripts several times to achieve familiarity with the content of the interviews. A coding structure was developed reflecting the key overarching topic areas that needed to be addressed in order to answer the research question (knowledge, awareness, attitude and behaviour). However, as the interview coding process was undertaken, inductively generated codes on new data themes were added. Agreement of themes was reached through discussion with a further researcher (EO). Data saturation was achieved as no new themes emerged after seventeen interviews [15].

Once coding was complete, findings were inputted into the Health Belief Model (HBM) framework. This was to better understand how desired actions could be achieved around HBV knowledge, attitude and practice in the student population. The HBM suggests that an individual’s action is influenced by their beliefs and their ability to act on those beliefs [17]. The six major domains are: perception of the benefits of acting, perception of the barriers to acting, perception of the severity of the disease or outcome, perception of the individual’s susceptibility to the disease or outcome, cues to act and self-efficacy. Self-efficacy is the sense of being able to act on a decision; a core component being the belief that the individual is “in control of their own behaviour” [18]. The identified desired actions were: safe sex, safe piercing and tattoo practices, and seeking HBV testing and vaccination if required. We acknowledged that preventing HBV was one of many health promoting aspects of safe sex and safe piercing and tattoo practices and our model would be restricted to perceptions around HBV.

## Results

Table 1 details the demographic characteristics of the twenty students who were interviewed. Four major themes were identified which are discussed below and supported by quotations. For participants from outside of the UK, their specific countries of origin are not included in order to maintain confidentiality.

**Table 1:** Participant demographics

Demographic	Category	Number (%)
Gender	Male	9 (45)
	Female	11 (55)
Level	Undergraduate	5 (25)
	Postgraduate	15 (75)
College	Arts & Social Sciences	5 (25)
	Life Sciences & Medicine	11 (55)
	Physical Sciences	4 (20)
Nationality (by WHO Region)	UK	4 (20)
	Europe/Central Asia	9 (45)
	Sub-Saharan Africa	5 (25)
	South Asia	1 (5)
	East Asia/Pacific	1 (5)

### Theme 1: knowledge and awareness

In general, participants reported feeling like they had low levels of knowledge about HBV. Participants often talked about Hepatitis A, B and C together and there was frequently confusion over the difference between them:

“*I know that there are three types I think and I don’t know the differences*.” (Female, postgraduate, Western Europe).

It was only those who had a friend or family member affected by HBV who felt they ever considered the disease; the majority of participants rarely did. Emphasis rested on knowledge that a vaccination was available and that the virus could be transmitted through blood. Unprotected sex and intravenous drug use were highlighted as risk factors. Signs and symptoms of HBV were infrequently discussed; the main ones mentioned being yellowing of the eyes and skin. Eastern European and Sub-Saharan African participants appeared to associate HBV with these signs to a greater degree than the British participants:

“*I know that usually people who have Hepatitis their skin becomes yellow and this disease affects the liver.*” (Male, postgraduate, Eastern Europe).

There was confusion over the specifics of how the virus is physically spread through infected blood or bodily fluids. For example, a participant who works in hospital wards described how he lacked clarity in how transmission occurs:

*“I don’t know if you can pass it on if you’re on the wards obviously and if you do come in contact with a patient who has got Hepatitis B.”* (Male, undergraduate, UK).

There was further lack of clarity over the HBV test; from what the test involved to how to interpret the results. More specifically, there was confusion around whether the antibody level checked post-vaccination is the same as the test, as highlighted by this student:

*“I mean I think I got my first vaccination in [year] and then I had one of these tests to see whether all the antibodies are still reactive and then, as a result, I was given a booster... So is that the test? What kind of test are you referring to?”* (Male, undergraduate, Eastern Europe).

Formal education had a very limited role in providing knowledge except for those undertaking a medical degree. Awareness of HBV, but not necessarily knowledge levels, had previously been raised transiently amongst some participants through posters, newspaper articles, television programmes and face-to-face public health campaigns. Most emphasis in fact was placed on obtaining the HBV vaccination and the knowledge that was acquired during that process. The HBV vaccination was sought for a number of reasons including vaccination requirements prior to starting a new job, travel, as part of the routine childhood programme in their home country, following health professional advice due to being identified as part of an at risk group and because family members had been vaccinated. Despite obtaining some knowledge through the vaccination process, it was still acknowledged to be limited:

“*I also went abroad quite a bit, so I made sure that I had all my vaccinations. But, you see like at the moment, I couldn’t even tell you if it is up to date or if I would have to get a second one at some point.*” (Female, postgraduate, Western Europe).

### Theme 2: attitudes and views

The words ‘serious’, ‘dangerous’, ‘deadly’, ‘terrible’ and ‘horrible’ were used to describe the disease. The concept of seriousness tended to be linked with the idea of it being incurable. There was only one participant who suggested it was not a serious condition and in this case, this was also associated with a difference in how the student viewed the length of time that someone would have HBV for, stating that you do not have HBV for a long time. There was vague acknowledgement that being told you had HBV would change your life. Few went into any further details; one suggested that it would affect your studies and another that it would affect your family. Yet, others suggested that actually it’s not an important disease for them personally. As one participant put it:

*“It’s kind of just in that sea of things that I never cared about.”* (Female, undergraduate, Asia).

In fact, there was an extremely low perceived probability that the individual participants could contract HBV. The perceived prevalence of HBV in the UK was extremely low:

“*I think in the UK it’s been eradicated quite a while ago*.” (Male, undergraduate, Eastern Europe).

The majority also viewed themselves as outwith any high risk group and so therefore did not see themselves as at risk at all. This also likely reflects how students are mostly a young, well population and, alongside this, may reflect a perception of immunity to disease, as suggested by one of the participants:

*“I think that one of the problems with people our age is that, and myself included, is that we always feel that it’s for other people to have the diseases; like we are invincible because we are still young and healthy.”*(Female, postgraduate, Western Europe).

Those who acknowledged that they saw potential for being in contact with people who had HBV, for example healthcare workers, demonstrated that they also did not see themselves as at risk because they had been vaccinated:

*“I’ve had boosters. So, as far as I’m concerned, that’s dealt with; I don’t need to think about it.”* (Male, postgraduate, UK).

It was recognised that you must firstly be aware of a condition to have a view and since awareness of HBV is so low in general, this restricts associated attitudes towards it. This appeared to also limit stigma; in general prominence was placed on the idea that there is not a marked characterisation of the type of person who gets HBV. Exceptions to this were that it was associated with ‘dirty blood’, drug use, alcoholism and multiple sexual partners. However, even these associations did not necessarily equate to stigma, as reflected in the following quote:

*“People might assume it’s more to do with alcoholism and things like that...But then being a drinker is not so stigmatised; in fact in some parts of the UK it’s more celebrated.”* (Male, postgraduate, UK).

HBV was not viewed by the participants in isolation but in the wider context of disease. Those who had conversed with healthcare professionals about HBV tended to do so as part of a broader discussion around health, for example, at sexual health checks. Some participants acknowledged HBV in the context of sexually transmitted infections but stressed that it is not one commonly discussed:

“*In the context of like sexual relations and sexual behaviour, I don’t know whether there is any stress put on Hepatitis B; that it is generally a sexually transmitted disease. Like people don’t really generally mention that*.” (Female, postgraduate, Western Europe).

It was viewed differently to:

*“your regular STDs that everyone knows about.”* (Male, undergraduate, UK).

Furthermore, HBV was frequently compared to HIV. The diseases were viewed as similar in many respects; who is at risk, transmission routes, the seriousness of the conditions and that there is no cure. However, knowledge, awareness and the perceived global importance placed on addressing HBV were seen to be a lot lower than with HIV:

“*I guess other blood transmitted diseases get more attention. Such as HIV, everybody knows about it. Hepatitis B is, I think, no-one really is interested*.” (Male, undergraduate, Eastern Europe).

#### Theme 3: how knowledge, awareness and attitudes link to behaviours

Emphasis rested on the lack of influence HBV has on the participant’s behaviours, which was thought to be linked to low knowledge and awareness:

*“I wouldn’t say that I do anything with having any consciousness about Hep B and my actions at all.”* (Female, postgraduate, Western Europe).

Some participants proposed that even if people did acquire a higher knowledge around this subject, it would not lead to changes in behaviour:

*“I think people still participate in risky behaviour, even if they do know the consequences.”* (Female, postgraduate, UK).

Those who had been vaccinated were content to have limited knowledge on the disease. In contrast, those who had a friend or family member affected by HBV were motivated to do personal research in order to learn more, as highlighted by this participant:

*“I didn’t really bother to know about it but later on I realised [a family member] had got Hepatitis B and, I mean, I decided to read about it.”* (Female, postgraduate, Africa).

In general, the participants felt that it would be appropriate for there to be further HBV awareness raising initiatives but did not express a desire for in-depth knowledge. There was the feeling that generic information alone does not provide relevance to them at an individual level and that behaviour change is more likely to happen if the person perceives themselves as susceptible or knows someone close to them that is affected. This was supported by case stories where a change in personal risk assessment triggered action. One participant actively sought vaccination after learning she was to encounter HBV positive people as part of her work. Another sought to be tested after discovering a relative had the condition. In contrast, a different participant chose to cope with the possible risk by avoiding the issue and trying to forget about it. This highlighted the notion that people have different thresholds for action, even if they have the same risk. Females generally appeared to have lower thresholds for action than males. For example, a couple of participants, both females from non-UK countries, commented how they proactively seek assurance that dental equipment is clean prior to any oral checks:

“*I still try to make sure that if I am going, for example, to the dentist, to ask again whether everything was clean. I know it’s a little bit annoying. I mean it might hurt the person who is doing all this stuff but still, it’s just to me to be sure that it’s going to be ok*.”(Female, postgraduate, Asia).

Participants were more likely, and speculated that others were more likely, to take action if the action itself was deemed simple and not associated with social judgment. Vaccination was repeatedly seen as a simple action that many of the participants had undertaken. However, lifestyle was viewed as difficult to change. One participant suggested that people prefer to get vaccinated so that they do not need to change their behaviours:

*“People are now thinking, okay, let me go, if I can vaccinate then it’s better to vaccinate and then I continue to live in my careless lifestyle.”* (Female, postgraduate, Africa).

A further participant alluded to the idea that there was not one single trigger that led her to have a sexual health check (which included HBV testing) but multiple. The student discussed various prompts throughout the interview, for example:

*“I remember when I went to [name of place], in the toilet - if you don’t mind me talking about it? - in the toilet room, there was a leaflet about the Sexual Health Clinic which is one of the ways I found out about it.”* (Female, undergraduate, Asia).

### Theme 4: engagement with healthcare

Almost unanimously, the first place where participants sought health information was from the internet. Some were happy to use potentially unreliable sources whereas others would make a conscious effort to check that the information was from a perceived trustworthy source such as a university or NHS webpage. If further or more personalised information was required, they would then visit their General Practitioner (GP). Other points of contact suggested were the NHS health points, friends who work in healthcare and the library. Pharmacies and sexual health clinics were mentioned by participants who already had an established relationship with these services.

Their key considerations around whether to approach a healthcare professional to discuss HBV were: fear of knowing test results, the acceptability of the test and ensuring confidentiality.

*“I would prefer to go somewhere where I wouldn’t necessarily know the person that was testing me.”* (Female, postgraduate, UK).

Many international students compared the UK healthcare system to their system at home to highlight the differences and potential frustrations with the UK one. For example, some were frustrated at having to book an appointment and wait days to be seen rather than turn up to a clinic and be seen that day. Others had to adapt to GPs acting as a gatekeeper in the UK when they had been used to having direct access to specialists:

*“It’s very difficult to see a specialist here. So it took me some time to really start trusting a doctor that they know what they are doing, because they really are responsible for a variety of conditions in the first place.”* (Female, postgraduate, Western Europe).

Participants suggested various methods of communication for healthcare staff to engage with students; through social media, posters, during university induction most notably at fresher’s fair stalls, university based seminars and the student portal. Leaflets alone were not regarded as an effective way of reaching students. However, some suggested that they could be used to facilitate face-to-face discussions:

*“I feel that nobody actually looks at them [leaflets], unless someone is actually giving it to you and talking through it.”* (Female, postgraduate, Western Europe).

The effectiveness of posters split opinion but their location appeared important; on the back of toilet doors or in GP waiting areas were preferable as opposed to in university corridors:

“*Quite often students just don’t pay attention on what is on walls or in the corridor because they are too busy speaking to each other*.” (Male, postgraduate, Eastern Europe).

Those who engaged regularly with certain services highlighted the potential for these staff to raise awareness around HBV. This included pharmacists, sexual health staff and GPs:

*“I have a year appointment because of the birth control pill, so I’m assuming that that’s not considered protected sex and you need to use a condom to be protected of Hepatitis B. So probably in those types of appointment, it would be nice to discuss it because I’m not sure that the patient would ever bring it up if the patient is not aware it is a risk?”* (Female, postgraduate, Western Europe).

The participants wanted any information provided on HBV to be short, straightforward and accessible. One participant suggested using the student portal:

“[on the] *student portal...there should be something on maybe your health and there’s different things; physical, emotional, blah, blah, blah. And maybe something on STDs, Hepatitis, that they can easily click on and have a PDF summary sheet and then it gives them an idea of if you’re seeing this, if you’re seeing that, these are the numbers to call or the people to see*.” (Male, postgraduate, Africa).

They also highlighted that it should be delivered in an interactive manner:

“*I’ve been taught about but it’s not necessarily something that I had to process or something that I had to think about and talk about it. I think there is a subtle difference when you have to do that*.” (Male, undergraduate, Eastern Europe).

Finally, they valued personalised over generic information and emphasised that it should be clearly relevant to the student population:

“*You put a face on a disease, especially if it’s someone that’s as young as you are, it makes it harder to look the other way; it makes it easier to relate to it*.” (Female, postgraduate, Western Europe).

## Discussion

### Key findings and the health belief model

This study found students in Aberdeen, North East Scotland, to have low levels of knowledge and awareness of HBV. Knowledge was stronger around basic transmission routes and the presence of a vaccine. Symptoms, testing and treatment were less well understood. This is in keeping with survey results of students in Ethiopia and Iran [12, 13]. The Ethiopian survey also demonstrated that only 50% of the trainee health care professionals were adhering to good HBV preventative practice when working with patients, suggesting that knowledge of transmission routes alone is not enough [12]. A further survey in Morocco indicated that, despite 87% of dental students knowing that HBV could be contracted through occupational blood exposure, only 40% took any action when they were personally exposed [19]. Our study revealed students to have a very low perceived risk of contracting HBV, which could partly explain the gap between knowledge and practice. Previous studies have demonstrated low perceived risk in the student population regarding HIV [20] and Human Papillomavirus (HPV) infection [21, 22].

**Fig. 1:**
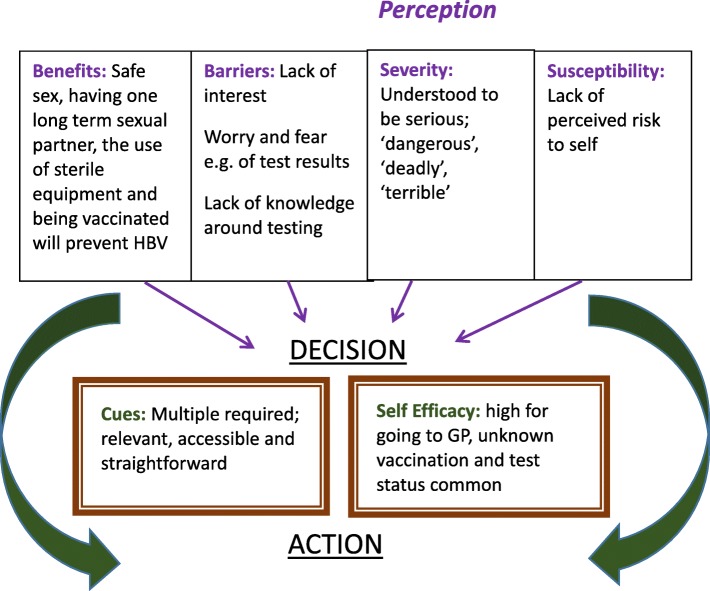
Key findings within the HBM framework

We used the Health Belief Model (HBM) as an explanatory framework to better understand how the following actions could be promoted in the student population: safe sex, safe piercing and tattoo practices, and seeking HBV testing and vaccination if required (Fig. 1).

Vaccination, use of sterile equipment at tattoo parlours, safe sex and having one long-term sexual partner were perceived to be effective ways of preventing HBV. Participants were particularly positive about vaccination, viewing this as a simple action which was free from stigmatisation. However, a study looking at predictors of HPV vaccination uptake rate in young American women found that the level of perceived benefit was not associated with uptake [23]. The key barrier to action was the students’ lack of perceived susceptibility to HBV. Other barriers included lack of interest in obtaining in-depth knowledge, difficulty in changing lifestyle habits, worry regarding asking about professional needle practices, lack of knowledge around testing and fear of test results.

This study indicates that multiple cues in different formats are required to increase the likelihood of action. Information for students should be tailored to their particular needs, be easily accessible, clear and short. A combined Health Belief and Theory of Planned Behaviour Model for HPV vaccination demonstrated self-efficacy to be another important factor. This is consistent with the findings of Koch et al. who showed self-efficacy to be key in an individual acting on their desire to get a tattoo [18]. In our study, we found self-efficacy to be high for approaching GPs and pharmacies. Tanya et al. identified that the HBM predicted condom use in American college students to a greater extent when the roles of peer norms and alcohol were included [24]. We did not specifically look at these in the context of HBV-related behaviours but could be included in future research on this topic.

### Strengths and limitations

This study has explored an under-researched topic and in doing so, helps to address this gap. From our knowledge, this is the first study to look at the HBV knowledge, awareness and associated behaviours of university students in the UK. The qualitative study design allows rich data to be captured and the ability to look at possible explanations for why desired actions regarding HBV prevention are not achieved. We were unable to get participants to review the study findings. However, the findings are supported by participant quotations, their alignment with previously published quantitative survey results and consistency with local healthcare provider experience. Data saturation also provides a level of reassurance that all major themes were identified.

Due to the nature of how we had to recruit participants, the study relies on convenience sampling at one university. Despite this, participants varied in gender, nationality, degree and whether they were at undergraduate or postgraduate level. It is not possible to fully eliminate the potential risk of selection bias as volunteers were self-selecting and therefore more likely to have had extremes of opinion. Yet, our findings suggest any bias to be minimal since participants generally lacked in-depth knowledge and did not see HBV as a disease directly relevant to them. The data was collected by a researcher with no previous associations with the students or HBV services. This may have had advantages such as greater opportunity for honest participant-directed interviews and lack of researcher bias in data analysis. On the other hand, it may have limited the depth of interview discussions. Finally, the interviews were conducted after the online surveys were completed. It is possible that levels of HBV knowledge and awareness were increased as a result of completing the survey. However, the aim of the interviews was not to quantify knowledge and awareness but rather to explore the perspectives of students. When asked directly about where their knowledge had come from, the participants did not associate this with completing the survey.

## Recommendations

Overall, we suggest that there is a need for more targeted HBV messages with clearer risk communication to the student population. These should highlight the main risks for contracting HBV for students – for example unprotected sex, and getting tattoos and piercings abroad. Our study also indicates that further clarity is needed for students around when to get tested and what this entails.

How these messages are delivered is extremely important. Our findings suggest that it may be helpful to put HBV messages in the context of sexual health. HBV information, therefore, should be further incorporated into sexual health promotion and materials. Leaflets may be more effective if they are used as a basis for discussion with a member of staff rather than on their own. Consideration also needs to be made around how to make information as accessible as possible to students. This could include the use of student portals, social media platforms and relevant websites.

Further work is required to look at the best ways of improving student self-efficacy in terms of encouraging individuals’ sense of control over preventative behaviours.

## Conclusions

Knowledge and awareness of HBV was generally low in the university students interviewed in Aberdeen, North East Scotland. Although it was acknowledged as a serious disease, the students did not perceive themselves to be at risk and rarely acted in a manner that took HBV into consideration. There is a need for targeted information aimed at students which is simple and delivered in an accessible and interactive way.

## Abbreviations

**GP**: General Practitioner
**HBV**: Hepatitis B virus
**HIV**: Human immunodeficiency virus
**HPV**: Human Papillomavirus
**STD**: Sexually transmitted disease
**UK**: United Kingdom
